# Does specialist physician supply affect pediatric asthma health outcomes?

**DOI:** 10.1186/s12913-018-3084-z

**Published:** 2018-04-05

**Authors:** Guido Filler, Tom Kovesi, Erik Bourdon, Sarah Ann Jones, Laurentiu Givelichian, Cheryl Rockman-Greenberg, Jason Gilliland, Marion Williams, Elaine Orrbine, Bruno Piedboeuf, The Paediatric Chairs of Canada Mark Bernstein, Robert Connelly, Denis Daneman, Ciarán Duffy, Marc-Andre Dugas, Allison Eddy, Guido Filler, Jean-Yves Frappier, Susan Gilmour, Laurentiu Givelichian, Cheryl Rockman-Greenberg, Lennox Huang, James D. Kellner, Michael Shevell, Cathy Vardy, Hervé Walti

**Affiliations:** 1 Departments of Peediatrics, Children’s Hospital at London Health Sciences Centre, University of Western Ontario, 800 Commissioners Road East, London, Ontario N6A 5W9 Canada; 2 Departments of Medicine, Children’s Hospital at London Health Sciences Centre, University of Western Ontario, London, Ontario Canada; 3 Departments of Pathology & Laboratory Medicine, Children’s Hospital at London Health Sciences Centre, University of Western Ontario, London, Ontario Canada; 4 Department of Pediatrics, Children’s Hospital of Eastern Ontario, University of Ottawa, Ottawa, Ontario Canada; 5 The Canadian Institute for Health Information (CIHI), Ottawa, Ontario Canada; 6 Departments of Surgery, Children’s Hospital at London Health Sciences Centre, University of Western Ontario, London, Ontario Canada; 7 Department of Pediatrics, University of Saskatchewan, Saskatoon, Saskatchewan Canada; 8 Department of Pediatrics and Child Health, University of Manitoba, Winnipeg, Manitoba Canada; 9 Department of Geography, University of Western Ontario, London, Ontario Canada; 10 Paediatric Chairs of Canada (PCC), Ottawa, Ontario Canada; 11 Department of Pediatrics, University Laval, Faculty of Medicine, Quebec City, Quebec, Canada

**Keywords:** Health manpower/trends, Physicians/supply and distribution, Pediatric, General practitioners

## Abstract

**Background:**

Pediatrician and pediatric subspecialist density varies substantially among the various Canadian provinces, as well as among various states in the US. It is unknown whether this variability impacts health outcomes. To study this knowledge gap, we evaluated pediatric asthma admission rates within the 2 Canadian provinces of Manitoba and Saskatchewan, which have similarly sized pediatric populations and substantially different physician densities.

**Methods:**

This was a retrospective cross-sectional cohort study. Health regions defined by the provincial governments, have, in turn, been classified into “peer groups” by Statistics Canada, on the basis of common socio-economic characteristics and socio-demographic determinants of health. To study the relationship between the distribution of the pediatric workforce and health outcomes in Canadian children, asthma admission rates within comparable peer group regions in both provinces were examined by combining multiple national and provincial health databases. We generated physician density maps for general practitioners, and general pediatricians practicing in Manitoba and Saskatchewan in 2011.

**Results:**

At the provincial level, Manitoba had 48.6 pediatricians/100,000 child population, compared to 23.5/100,000 in Saskatchewan. There were 3.1 pediatric asthma specialists/100,000 child population in Manitoba and 1.4/100,000 in Saskatchewan. Among peer-group A, the differences were even more striking. A significantly higher number of patients were admitted in Saskatchewan (590.3/100,000 children) compared to Manitoba (309.3/100,000, *p* < 0.0001).

**Conclusions:**

Saskatchewan, which has a lower pediatrician and pediatric asthma specialist supply, had a higher asthma admission rate than Manitoba. Our data suggest that there is an inverse relationship between asthma admissions and pediatrician and asthma specialist supply.

## Background

There is substantial variability in the ratio of pediatric subspecialists to the population of children among the Canadian provinces, with higher ratios in provinces such as Alberta (AB) and Manitoba (MB), and lower ratios in provinces such as Saskatchewan (SK), British Columbia (BC) and Ontario (ON) [1]. In some provinces, the physician density in certain subspecialties can be up to four times greater than in others [1]. The cause of the uneven distribution of physicians is multifactorial, and an imbalanced physician distribution has been identified as one of the major challenges facing the healthcare workforce.

Regional variability of access to pediatricians and pediatric subspecialists results in prolonged wait times [2, 3]. Best practice-based patient management may greatly influence asthma hospitalization inpatient rates and emergency visits [4]. Expert-based asthma care may be more likely to follow consensus guideline recommendations [5]. A potential consequence of not having access to asthma specialists may be reduced patient and family access to asthma education and lack of such access has been shown to worsen childhood asthma outcomes [6].

There is evidence that expert-based outpatient asthma care improves asthma outcomes in adults, including hospitalization rates and the level of asthma control [5, 7]. Data from Manitoba (MB) suggest the reported prevalence of diagnostic label of asthma in adults is positively related to the number of referrals to specialists [8]. In the world of pediatric asthma, the effect that access to expert care has on health outcomes is unknown. We hypothesized that a paucity of pediatricians and pediatric asthma specialists (pediatric pulmonologists and allergists [9]) would lead to worse health outcomes. In this novel study, we elected to compare asthma health outcomes within comparable health regions in Saskatchewan (SK), the province with the lowest supply of pediatric asthma subspecialists, with MB, the province with one of the highest supplies of pediatric asthma subspecialists. These provinces were also selected due to their similarities in climate, population diversity, indigenous populations, housing conditions, and other variables (see Table 3). We hypothesized that the differing supply of pediatricians and pediatric asthma specialists would affect admission rates.

## Methods

Inter-regional comparisons of pediatric asthma hospitalizations should control for the multiple factors that may influence asthma prevalence and severity, including demographic, environmental, and socio-economic factors [7]. Unfortunately, a multivariate analysis could not be performed as CIHI privacy regulations prevent sharing of record-level information. Therefore, a multistep approach was used instead, to allow regional comparisons while attempting to control for other relevant factors, as follows:

i. Within provinces, health regions have been defined by each provincial government, and are termed Regional Health Authorities (RHA). At the time of the analysis, there were 11 health regions in MB (2 of which are considered or include urban areas) and 13 in SK (2 of which are urban).
ii. Health region peer groups have been defined by Statistics Canada based on 24 variables that are important determinants of health, including a variety of socioeconomic, social, and demographic factors. Using these defined peer groups, it is possible to compare regions with similar socio–economic characteristics. Statistics Canada performed a stepwise discriminant analysis and found that, of the 24 variables, 4 were found to play a key role in defining the health region peer groups: population density, proportion of indigenous population, proportion of immigrants, and employment rate [10].
iii. Due to privacy considerations, rural peer groups could not be examined because the small number of children meeting study criteria in sparsely-populated peer groups (< 5 entries per peer group) would make these children potentially identifiable.
iv. For our analysis, we therefore used patient data originating from peer group “A,” which includes 4 predominantly-urban health regions grouped together based on their similar social and economic health determinants (Saskatoon RHA and Regina Qu’Appelle RHA in SK, and Brandon RHA and Winnipeg RHA in MB). Peer Group A is distinguishable from the other Peer Groups because the values for each of the 4 defining variables fall within the 35th percentile to 65th percentile range (the medium range) [10].
v. We compiled a geo-database of all family physicians with their postal code using the Canadian Institute of Health Information (CIHI) – Scott’s Medical Database. Each record was associated to a dissemination area using the postal code and Statistics Canada’s Postal Code Conversion File (PCCF) and then geo-coded. Pediatrician and pediatric subspecialty data were obtained from validated academic & provincial databases provided by the Pediatric Department Chairs in both MB and SK. Asthma experts were defined as pediatric allergists and pediatric pulmonologists [9]. The provincial physician workforce (general practitioners (GPs) and pediatricians) was then mapped for each province and health region, using maps generated by ArcGIS 10.1, a commercially available geomapping software program.
vi. Since peer groups have similar characteristics, mapping the physician workforce in each health region allowed us to assess the impact of the workforce on patient outcomes for asthma. This assessment was relatively independent of the other parameters that could affect asthma morbidity and health outcomes [7].
vii. Using the Canadian Association of Pediatric Health Centers - Pediatric Decision Support Network (CAPHC-CPDSN) database, data extraction from the CIHI Portal [FY2009–2010 DAD]), the Case Mix Group (CMG) [11] comprising pediatric admissions to hospital for upper/lower respiratory was identified and data for CMG 147 (asthma) were extracted. The following parameters were collected:
  - Total number of hospital admissions in the provinces of MB and SK,
  - Length of stay (LOS) [12],
  - Expected length of stay (ELOS) [12]. The calculation for ELOS as defined by Statistics Canada: (a) takes into account the reason for hospitalization, age, comorbidity, and complications; (b) uses case mix group methodology (statistically and clinically homogeneous groups based on the collection of clinical and administrative data); and (c) is calibrated for the given year [13]. ELOS and LOS were compared to determine whether the actual LOS was longer or shorter than predicted, based on disease severity.
  - Case Mix Group Resource Intensity Weight. Case Mix Groups are defined by CIHI, and combine inpatients with similar resource-use characteristics. The Resource Intensity Weight describes total patient resource use, for a particular case mix group [12].
viii. Provincial-level health outcomes were compared with physician manpower per 100,000 children.
ix. Asthma admission rates are affected by a number of variables, including age [14], climate [15, 16], ethnicity [7, 17], sex [14, 18], housing [19], income [19], environmental tobacco smoke exposure [16], and many other factors. We analyzed these covariates within the peer-group comparison.

### Statistical analysis

The data analysis was performed directly from the Excel spreadsheets provided by each centre. The actual admissions were provided through the CIHI portal. Population was based on the 2011 census, and data for 2010 and 2012 were estimated based on population growth. Statistical analysis was performed with simple statistical tests using Excel (Microsoft ® Excel for Mac version 12.1.0 (080409) and GraphPad Prism version 4.02 for Mac (GraphPad Software, San Diego, CA, U.S.A.). Continuous data were analyzed for normal distribution with the Shapiro Wilks test. Since data were normally distributed, they were reported as mean and standard deviation. Admission rates were compared using the Fisher’s exact test. Administrative data were compared using unpaired t-test. A *p*-value of < 0.05 was considered statistically significant.

## Results

**Fig. 1:**
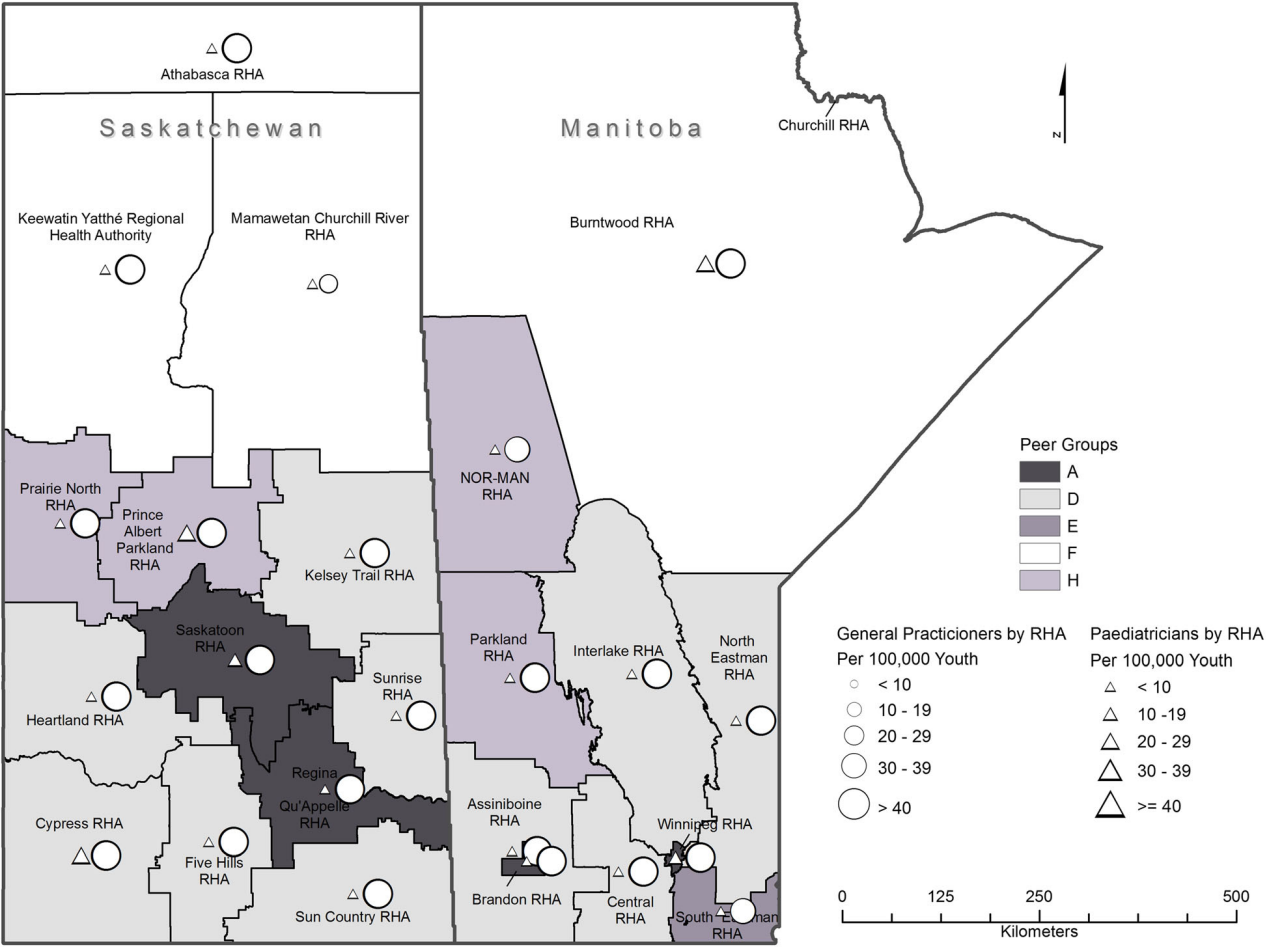
Density of paediatricians and general practitioners in the provinces of MB and SK based on health region. RHA: Regional Health Authority (Health Region). Statistics Canada defines a RHA as an administrative area defined by the provincial ministries of health. The figure was generated by JG, based on a shapefile provided by EB

The populations of the two provinces were comparable (MB 1,208,268; SK 1,053,960). MB had 48.6 pediatricians and pediatric subspecialists per 100,000 children compared to 23.5 in SK. Figure 1 maps all general practitioners and pediatricians in both provinces, according to health regions, with health regions of the same peer group shaded identically. In MB, 3 cities: Winnipeg, Brandon and Thompson had pediatricians, whereas in SK pediatricians were located in 5 cities: Regina, Saskatoon, Prince Albert, Swift Current, Moose Jaw, Yorkton and North Battleford. Province-wide, there were 1.1 pediatric pulmonologists per 100,000 children in MB and 0.4 in SK respectively. Moreover, there were 2 pediatric allergists in MB per 100,000 children and 1 in SK. With regards to peer group comparison, there was a much higher pediatrician and pediatric subspecialist density in the peer group “A” cities of Winnipeg and Brandon, MB when compared to Regina and Saskatoon, SK (Fig. 1, Table 2).

At the provincial level, there were 967 admissions for asthma in MB and 1533 in SK (CMG 147) in the year 2011. The number of admissions /100,000 child population were 309.3 in MB and 590.3 in SK (*p* < 0.0001, Fisher’s exact test). Table 1 provides characteristics of patients in both provinces. The mean actual LOS was not significantly different: 2.7 versus 3.1 days (*p* > 0.5). The sum of the acute LOS was 3014 days in MB versus 4153 days in SK, a difference which was not statistically significant. The patients in MB tended to be sicker; their ELOS was 2.9 days in MB versus 2.4 days in SK, although the difference was not statistically significant. Similarly, Case Mix Group-based Resource Intensity Weight was very similar in both provinces.

**Table 1:** Admissions for asthma in children and youth under 18 years of age in the two provinces. Data were based on the 2011 census and the 2011 calendar year. LOS = length of stay; ELOS = expected length of stay

Parameter	MB	SK
Total population	1,208,268	1,053,960
Child population	312,598	259,709
General practitioners /100,000 child population	386.4	383.1
General paediatricians /100,000 child population	19.2	10.0
Specialized paediatricians (all subspecialties) /100,000 child population	29.4	13.5
Number of admissions	967	1533*
Number of admissions /100,000 child population	309.3	590.3*
Number of readmissions	181	152
Number of readmissions /100,000 child population	57.9	58.5
Total acute LOS	3014	4153
Average ELOS	2.9 days	2.4 days
Average LOS	3.1 days	2.7 days

* p < 0.0001, Fisher’s exact test

Table 2 provides a comparison of the four health regions belonging to peer group A. Similar to findings by province in Table 1, the number of pediatric asthma cases /100,000 child population was significantly higher in peer group A regions in Saskatchewan (215.8/100,000) than in the same peer group in Manitoba (62.4/100,000) (*p* = 0.012) from 2010 to 2012.

**Table 2:** Comparison of key administrative data as cases/100,000 child population in the four peer-group A health regions: Case mix group plus rate intensity weight using case mix group and rate intensity weight (CMG + RIW); expected length of stay (ELOS); and acute length of stay (LOS); not significant (n.s.). Actual cases were taken from the CIHI database. Population was extrapolated based on 2011 census data and the annual growth rates. Cases/100,000 child population were calculated from real numbers. The 3 right columns were provided from the CIHI portal. In the bottom of the table, the averages for the 3 years are provided

Province	Peer Group A Regional Health Authority	Year	Cases	Population < 19	Cases / 100,000 child population	CMG/RIW (Average)	ELOS Days (Average)	Acute LOS Days (Average)
Manitoba	Winnipeg	2010	79	167,557	47.1	0.57	1.9	2.2
	Winnipeg	2011	88	169,592	51.9	0.46	1.8	2.2
	Winnipeg	2012	113	171,627	65.8	0.50	1.9	2.3
	Brandon	2010	17	13,714	124.0	0.41	1.8	2.1
	Brandon	2011	26	13,881	187.3	0.45	1.7	1.7
	Brandon	2012	21	14,048	149.5	0.41	1.7	2.0
Saskatchewan	Regina Qu’Appelle	2010	199	65,088	305.7	0.44	1.8	2.0
	Regina Qu’Appelle	2011	130	66,484	195.5	0.45	1.8	2.0
	Regina Qu’Appelle	2012	98	67,880	144.4	0.43	1.7	1.8
	Saskatoon	2010	55	22,516	244.3	0.40	1.7	2.1
	Saskatoon	2011	44	22,999	191.3	0.47	1.7	2.0
	Saskatoon	2012	51	23,482	217.2	0.51	1.9	2.5
Average over 3 years								
Manitoba	*Combined Manitoba Peer Groups A* *(Winnipeg RHA + Brandon RHA)*	*2010–2012*	115	183,473	62.4	0.47	1.8	2.08
Saskatchewan	*Combined Saskatchewan Peer Groups A* *(Regina* Qu’Appelle *RHA + Saskatoon RHA)*	*2010–2012*	192	89,483	215.8	0.45	1.77	2.07
Comparison between provinces (unpaired Student t-test)						n.s	n.s	n.s.
Comparison between provinces (Fisher’s Exact Test)					0.012*			

With regards to covariates affecting asthma admission rates, the cities in the four peer groups appear to be similar (Table 3), with only the percentage indigenous population and household income being significantly different (*p* = 0.02, Table 3). While higher admission rates have been found to be associated with lower income and indigenous ethnicity [20], incomes were lower, and the proportion of the population that is indigenous was higher, in MB. Outdoor air quality, defined using annual mean respirable particles (PM10) was very similar in Winnipeg, Brandon, Regina, and Saskatoon (11, 24, 21, and 12 mcg/m^3^, respectively (2010 Data) [21]. While previous Canadian research has related mean aeroallergen levels to asthma hospitalizations [15], mean aeroallergen counts were not available for SK cities, so this factor could not be compared. Population rates of smoking in MB and SK are extremely similar: 18.7 and 19.2% (2011 data) [22]. As a proxy for climate, we compared days above 0 degrees Celsius and growing days (defined as average temperatures above 5 degrees Celsius), and found that both of these variables were also similar in MB and SK. As sex and age affect patient outcomes [14], we also compared the patient demographics in the four peer group A health regions. The male to female ratio was comparable in both provinces (*p* = 0.538). Age was also not significantly different. The percentage of young children aged 1–4 was 63.8% in MB and 61.6% in SK (*p* = 0.599). When only using data on pediatric patients from the peer group A health regions, there was a clear inverse relationship between the number of pediatricians and subspecialists, and specifically pediatric asthma specialists, and the admission rate.

**Table 3:** Key environment (2012) and housing parameters (2011 Census, 2011 Household Survey) that may affect asthma admission rates in the four peer-group A health regions in MB and SK

Parameter		Winnipeg	Brandon	Regina	Saskatoon	t-test^a^
Days above 0 °C		175	171	168	170	0.11
Growing days (average temperature above 5 °C)		181	182	182	177	0.26
Average persons per household		2.4	2.3	2.4	2.4	0.21
Housing in need of major repair (%)		9.30	6.70	9.40	6.20	0.47
Indigenous population (%)		11.7	11.23	10.2	10.24	0.02
Immigrant population (%)		21.88	12.86	11.16	11.93	0.16
Population density per square kilometer		1430	599	1328	1060	0.36
Average income ($)		38,159	38,544	45,698	43,497	0.02
Prevalence of low income in 2010 based on after-tax low-income measure (%)^b^	< 18 years	22.8	20.9	18.6	18.5	0.04
Prevalence of low income in 2010 based on after-tax low-income measure (%)^b^	< 6 years	26.3	24.2	23.9	21.6	0.12

^a^comparing the two provinces; ^b^2010 data.

Sources: Environmental data from Environment Canada database (http://climate.weather.gc.ca/, last accessed on 17-Dec-2013), Stats Canada 2011 Census (http://www12.statcan.gc.ca/census-recensement/2011/dp-pd/index-eng.cfm, last accessed 3-Apr-2018), and Stats Canada 2011 National Household Survey (http://www12.statcan.gc.ca/nhs-enm/index-eng.cfm, last accessed 18-Dec-2013)

## Discussion

Our analysis demonstrated significant differences in the rate of asthma admissions between the provinces of MB and SK. Significantly more patients were admitted in SK, when compared with MB, and the patients in SK had a lower acuity but the same LOS. At the same time, there was a much lower density of pediatricians and pediatric subspecialists in SK when compared with MB. It appears that–similar to adults [7]–decreased access to pediatricians and pediatric asthma experts has a negative impact on admissions with asthma.

A variety of studies [17] have suggested that care by asthma experts improves asthma outcomes. Vollmer et al. evaluated 914 children and adults with asthma followed in an American health maintenance organization and found that patients managed by an allergist were more likely to take daily asthma medications and inhaled anti-inflammatory agents than patients followed by a generalist [5]. The allergists’ patients were also less likely to visit the emergency department and had higher quality of life scores [5]. Similarly, Wu et al. examined 1954 adults with asthma treated by a managed care company and found that patients treated by an asthma expert rather than a generalist were more knowledgeable about asthma, more likely to have received inhaled corticosteroids, less likely to have had emergency department visits or hospitalizations and had better asthma control [7]. Pediatric pulmonologists, pediatric allergists and pediatricians can provide expert asthma care [16]. Klomp et al. reported that suboptimal asthma control was common in SK and was associated with inadequate use of inhaled corticosteroids, including lack of use or inadequate doses [23]. As our findings suggest that disparities in access to pediatric asthma specialists were associated with an increased number of asthma hospitalizations in SK, we hypothesize that a significant number of admissions in SK may have been avoidable.

Our findings are particularly concerning given wide interprovincial variations in the national availability of adult and pediatric pulmonologists, as discussed by Cockcroft and Wensley in 2000 [24]. In fact, reported average wait times to see an adult pulmonologist in SK were 7–8 weeks for university-based consultants and 4–5 weeks for community-based consultants [24]. Given that the ratio of pulmonologists to the population was 1:86,000 for adults and 1:756,000 for pediatrics, it can be safely assumed that wait times are a great deal longer for pediatric patients. Given the disparity in the density of asthma experts in SK as compared to MB wait times can be assumed to be considerably longer in SK.

Our study is limited by demographic and environmental data, which can substantially affect asthma control, severity and admission rates. However, the four group A peer regions were remarkably similar and should thus have similar asthma incidence rates. Only indigenous population and average income differed slightly between the group A peer regions in both provinces. However, assuming the trends found in previous studies, the differences should have resulted in higher admission rates in MB. It should be emphasized that data required for a multivariate analysis could not be released due to privacy considerations, and fewer than 5 entries were omitted for the same reason. For this reason, we could only compare group A health regions with large populations.

The study is also limited by the assumption that non-subspecialists including pediatricians are not asthma “experts.” It is certain that many pediatricians and some family physicians have special expertise, experience, and interest in asthma care in children. Additionally, it is likely that some expert asthma care in SK and MB is provided by adult pulmonologists and allergists. Nevertheless, the number of these types of physicians is likely small and is therefore unlikely to have substantially impacted our findings. We base this on the low number of physicians with predominantly adult billings that billed for patients under 18. Asthma hospitalization rates and LOS are likely affected by numerous other factors, including age distributions, sex [14, 18], poverty [25], and ethnicity [26], including indigenous origin [20], but these parameters are similar in peer group A health regions and/or have been taken into account. Asthma misclassification is possible, although it has been shown that asthma classification in large Canadian databases is generally accurate [27]. Differences in access to expert physicians may have complex effects on asthma classification. In children, there is significant overlap between asthma and a variety of other conditions, including bronchiolitis, recurrent upper respiratory tract infections, and croup. Asthma experts may be more likely to diagnose asthma accurately. Conversely, asthma may be over-diagnosed by non-expert physicians, who may also be less reliant on objective testing in children 6 years and older, as recommended by current guidelines [28]. Our data are unable to determine whether SK has an increased number of shorter admissions as a result of unnecessarily early admission to hospital, or whether admission for children in MB is unduly delayed so children are sicker when they are admitted. Inaccuracy in the numbers of specialists and family doctors is always a possibility; however, we believe that the errors are negligible based on the fee codes used for mapping the physicians and the confirmation of the accuracy by the department chairs.

The question of the generalizability of our findings is important. The literature is scarce on the topic. We are only aware of one study from comparing the US states Georgia and North Carolina [29]. That study suggests similar evidence for the association between geographical variation in access to primary and asthma specialists and pediatric asthma outcomes [29]. Thus, the conclusions presented in this study are likely more broadly applicable beyond Canada.

In conclusion, this novel study demonstrated disparities in specialist and subspecialist availability likely have an adverse impact on asthma control in Canadian children and youth from the provinces studies. Efforts to ensure more uniform availability of pediatricians and pediatric subspecialists will likely improve health outcomes in asthma care.

To the best of our knowledge, this is the first study to find an association between access to specialized health care providers and health outcome parameters from administrative databases. Further research is required to establish this relationship between health outcomes and providers across the nation, which in turn will enable health care providers to rank admission rates in equivalent peer group health regions according to the supply of specialists and subspecialists. This may lead to a better understanding of the number of health care providers necessary per region to optimize pediatric health outcomes. To validate the findings of this pilot study and to assess whether the findings apply to other disease groups, we plan to conduct a Pan-Canadian study using the same geo-mapping and peer group methodology.

## Conclusions

This pilot study demonstrates a link between specialist and subspecialist supply and health outcomes. It may be possible to perform health human resource planning by matching the number of specialists and subspecialists with the administrative data used to determine what the appropriate supply for a given population should be.

## Abbreviations

AB: Alberta
BC: British Columbia
CAPHC-CPDSN: Pediatric Decision Support Network
CIHI: Canadian Institute of Health Information
CMG: Case Mix Group
ELOS: Expected length of Stay
GP: General Practitioner
LOS: Length of Stay
MB: Manitoba
PCCF: Postal Code Conversion File
RHA: Regional Health Authority
SK: Saskatchewan
