# Correction to: Does specialist physician supply affect pediatric asthma health outcomes?

**DOI:** 10.1186/s12913-019-4752-3

**Published:** 2019-11-27

**Authors:** Guido Filler, Tom Kovesi, Erik Bourdon, Sarah Ann Jones, Laurentiu Givelichian, Cheryl Rockman-Greenberg, Jason Gilliland, Marion Williams, Elaine Orrbine, Bruno Piedboeuf, The Paediatric Chairs of Canada

**Affiliations:** 1 Departments of Peediatrics, Children’s Hospital at London Health Sciences Centre, University of Western Ontario, 800 Commissioners Road East, London, Ontario N6A 5W9 Canada; 2 Departments of Medicine, Children’s Hospital at London Health Sciences Centre, University of Western Ontario, London, Ontario Canada; 3 Departments of Pathology & Laboratory Medicine, Children’s Hospital at London Health Sciences Centre, University of Western Ontario, London, Ontario Canada; 4 Department of Pediatrics, Children’s Hospital of Eastern Ontario, University of Ottawa, Ottawa, Ontario Canada; 5 The Canadian Institute for Health Information (CIHI), Ottawa, Ontario Canada; 6 Departments of Surgery, Children’s Hospital at London Health Sciences Centre, University of Western Ontario, London, Ontario Canada; 7 Department of Pediatrics, University of Saskatchewan, Saskatoon, Saskatchewan Canada; 8 Department of Pediatrics and Child Health, University of Manitoba, Winnipeg, Manitoba Canada; 9 Department of Geography, University of Western Ontario, London, Ontario Canada; 10 Paediatric Chairs of Canada (PCC), Ottawa, Ontario Canada; 11 Department of Pediatrics, University Laval, Faculty of Medicine, Quebec City, Quebec Canada

**Correction to: BMC Health Serv Res (2018) 18: 247**

**https://doi.org/10.1186/s12913-018-3084-z**

In the original publication of this article [1], the institutional author’s name needs to be revised from The Paediatric Chairs of Canada Mark Bernstein to The Paediatric Chairs of Canada.
